# The Influence of Curcumin on the Downregulation of MYC, Insulin and IGF-1 Receptors: A Possible Mechanism Underlying the Anti-Growth and Anti-Migration in Chemoresistant Colorectal Cancer Cells

**DOI:** 10.3390/medicina55040090

**Published:** 2019-04-03

**Authors:** Seyed Ahmad Hosseini, Hamid Zand, Makan Cheraghpour

**Affiliations:** 1 Nutrition and Metabolic Diseases Research Center, Ahvaz Jundishapur University of Medical Sciences, Ahvaz 6135715794, Iran; seyedahmadhosseini@yahoo.com; 2 Department of Cellular and Molecular Nutrition, Faculty of Nutrition Science and Food Technology, National Nutrition and Food Technology Research Institute, Shahid Beheshti University of Medical Sciences, Tehran 1981619573, Iran; hamid_zand@sbmu.ac.ir

**Keywords:** curcumin, insulin receptor, colorectal cancer, insulin-like growth factor-1 receptor, 5-fluorouracil

## Abstract

*Background and objectives*: Mounting evidence shows that curcumin, a bioactive substance originating from turmeric root, has anticancer properties. Additionally, curcumin prevents the migration and metastasis of tumor cells. However, the molecular mechanism involved in the anti-metastatic action of curcumin is not clear. Most studies have suggested that migration inhibition is related to curcumin’s anti-inflammatory properties. Curcumin possesses a regulatory effect on insulin and insulin-like growth factor-1 (IGF-1) receptors and signaling. Insulin signaling is one of the important pathways involved in tumor initiation and progression; therefore, we proposed that the anti-metastatic effect of curcumin may mediate the downregulation of insulin and insulin-like growth factor-1 receptors. *Materials and Methods*: Viable resistant cells resulting from treating SW480 cells with 5-fluorouracil (5-FU) were subjected to curcumin treatment to analyze the proliferation and migration capacity in comparison to the untreated counterparts. To test the proliferation and migration potential, MTT, colony formation, and wound healing assays were performed. Real-time polymerase chain reaction (RT-PCR) was performed to measure the mRNA expression of *insulin-like growth factor-1R* (*IGF-1R*), *insulin receptor* (*IR*), and *avian myelocytomatosis virus oncogene cellular homolog* (*MYC*). *Results:* Our findings showed that curcumin significantly decreased *insulin* and *IGF-1 receptors* in addition to *MYC* expression. Additionally, the downregulation of the insulin and insulin-like growth factor-1 receptors was correlated to a greater decrease in the proliferation and migration of chemoresistant colorectal cancer cells. *Conclusions:* These results suggest the possible therapeutic effectiveness of curcumin in adjuvant therapy in metastatic colorectal cancer.

## 1. Introduction

Colorectal cancer is the third most common cancer and cause of death from cancer worldwide [1]. Metastasis, drug resistance, and cancer recurrence are the major causes of colorectal mortality. Earlier studies have reported that the origin of most cancers including colorectal cancer has been attributed to the activation of various signaling pathways that are involved in proliferation, apoptosis, and metastasis [2]. Therefore, the development of new treatments that target a subpopulation of tumor cells involved in metastasis, which are resistant to conventional therapy, could be an effective step.

Curcumin is a polyphenolic pigment generated from the rhizome of *Curcuma longa* (turmeric root). This substance has been used as a traditional remedy for hundreds of years in Asian countries [3]. It has low solubility and bioavailability, therefore, it is difficult to provide enough evidence for the clinical advantages of curcumin [4]. However, accumulating evidence in the last decade has indicated that curcumin possesses several nutritional and pharmacological benefits including antioxidant, anti-inflammatory, and anticancer properties [5]. It prevents tumorigenesis and potently reduces tumor dissemination and metastasis. Many preclinical studies using several tumor cell lines have shown that curcumin inhibits the growth of cancer cells. Clinical studies also support the anti-inflammatory effect of curcumin in patients with colorectal cancers [6,7]. Curcumin has been shown to be a potent inhibitor of a master transcription factor regulating inflammation, NF-κB [8].

According to cancer stem cell (CSC) theory, a small subpopulation of immortal cells is responsible for tumor generation, heterogeneity, and relapse. Metastasis, as the major cause of cancer mortality, originates from CSCs [9]. Some evidence has shown that curcumin targets CSCs by influencing pathways involved in self-renewal including Wnt/β-catenin, Sonic Hedgehog (SHH), and Notch pathways [10]. Some researchers believe that the anti-inflammatory effect of curcumin is also the reason behind its anti-metastatic property.

Moreover, there is evidence that shows that insulin and insulin-like growth factor-1 (IGF-1) have a functional role in cancer progression and metastasis [11,12,13,14]. Insulin and IGF-1 signaling have a large similarity in action; hence, their ligands may recruit the receptors of each other [15]. Additionally, both receptors can activate similar intracellular pathways through similar mechanisms [16]. Inhibition of IGF-1 reduces cancer growth and metastasis in some cancers such as pancreatic tumors [17]; however, the role of insulin receptor downregulation in the inhibition of cancer progression has not been fully addressed. It has been shown that progression of colorectal cancer has a positive correlation with hyperinsulinemia in diabetic patients [18]. Insulin may induce *matrix metalloproteinase-2* (*MMP-2*) mRNA expression through IRS1/phosphoinositide 3-kinase (PI3K)/Akt and mitogen-activated protein kinase (MAPK) signaling in HCT-116 human colorectal cells [19].

Since curcumin regulates several points of the insulin signaling pathway and downregulates insulin receptors [20], we hypothesized that curcumin reduces the capacity of colorectal cancer growth and dissemination through the downregulation of insulin and IGF-1 receptors in a model of a chemoresistant colorectal cell line. To investigate the role of curcumin in the inhibition of metastasis and the growth of colon adenocarcinoma, SW480 cells were treated with 5-fluorouracil (5-FU), and the surviving chemoresistant viable cells were used to analyze the inhibitory effect of curcumin on proliferation and migration. Our results suggest a potential role for curcumin in the reduction of the proliferation and migration of resistant 5-FU-treated SW480 cells.

## 2. Materials and Methods

### 2.1. Chemicals and Reagents

Curcumin (1,7-bis(4-hydroxy-3-methoxyphenyl)-1,6-heptadiene-3,5-dione) was purchased from Santa Cruz (Santa Cruz Biotechnology, Santa Cruz, CA, USA). MTT [3-(4,5-dimethylthiazol-2-yl)-2,5-diphenyltetra-zolium bromide, a yellow tetrazole], DMSO (dimethyl sulfoxide), RNase, and propidium iodide were obtained from Sigma (Sigma, St. Louis, MO, USA). Dulbecco’s Modified Eagle’s Medium (DMEM), fetal bovine serum (FBS), and trypsin/EDTA were purchased from Gibco (Pittsburgh, PA, USA). RNX-Plus Solution and the First Strand cDNA Synthesis Kit were purchased from Sinaclon (Sinaclon, Tehran, Iran). Finally, 5-fluorouracil (5-FU) was provided by EBEWE PHARMA (EBEWE Pharma, Unterach, Austria).

### 2.2. Cell Culture

The SW480 (human colon cancer) cell line was purchased from the National Cell Bank of Iran (Pasteur Institute, Iran). Cells were maintained at 37 °C with 5% CO_2_ in DMEM containing 10% FBS, 1% (v/v) penicillin (100 units/mL), 1% (v/v) streptomycin (100 µg/mL), and 1% (v/v) glutamine. The medium was changed every other day, and cells were passaged once a week.

### 2.3. Cell Cytotoxic Analysis and Determination of IC50 Dose for 5-FU

Cell cytotoxicity and IC50 dose for 5-FU were detected using the MTT colorimetric assay [21]. Cells were seeded at a density of 5000 cell/well in a 96-well plate. After overnight incubation, the cells were treated in triplicate with different concentrations of curcumin (1, 5, 10, 15, 20, 25, 30, and 50 μM), 5-FU (0, 10, 50, 100, 150, 200, and 300 µM), and DMSO as the vehicle. After 72 h incubation, the medium was aspirated from the wells and 20 µL of MTT solution (5 mg/mL in PBS) was added to each well, then incubated at 37 °C, 5% CO_2_, for 5 h, and the formazan crystal was solubilized using 200 μL DMSO. The samples were read at 570 nm on a microplate reader (Bio-Tek Instruments, Winooski, VT, USA).

The percentage of cytotoxicity was calculated as = (100 – (OD at 595 nm of curcumin-treated cells/OD at 595 nm of control cells) × 100). IC50, the concentration which inhibited growth by 50%, was calculated from the linear equation ((50% − low percentage)/(high percentage − low percentage)) × ((high concentration − low concentration) + low concentration).

### 2.4. Flow Cytometric Cell Analysis

The effect of curcumin on cell cycle and apoptosis was determined by DNA content using cell cycle analysis. Cells were seeded into a 12-well culture plate (2 × 10^5^/cm^2^) and incubated with 20 µM curcumin, or DMSO as the vehicle, for 72 h. Next, cells were harvested, washed twice with PBS, and fixed in 70% ethanol overnight at −20 °C. The cells were washed with PBS, and treated with 0.5 µM/mL RNase and incubated for 30 min in 37 °C. Then, they were stained with 50 µg/mL propidium iodide for 30 min. Cells were analyzed with a FACScan flow cytometer (Becton Dickinson).

### 2.5. RNA Extraction and Real-Time PCR Analysis

Total RNA was extracted from SW480 and 5FU-SW480 using RNX-Plus Solution according to the supplier’s protocol. First strand cDNA was generated with 3 µg of the extracted RNA using a First Strand cDNA Synthesis Kit. Real-time PCR (RT-PCR) was performed using the StepOnePlus Real-Time PCR System (Applied Biosystems, Foster City, CA, USA) using a SYBR Premix Ex Taq II master mix. The PCR conditions for *MYC*, *IGF-1R*, *insulin receptor* (*IR*), and *hypoxanthine-guanine phosphoribosyl transferase* (*HPRT*) mRNA were 20 s at 95 °C for denaturation, 20 s at 60 °C for annealing, and 30 s at 72 °C for extension, for a total of 40 cycles. The slope of a linear regression model was applied to determine the real-time PCR efficiencies for the target and internal control genes. In order to assess the PCR efficiencies, the threshold of the cycle (CT) to a specific threshold for a serial dilution of cDNA was measured. The efficiency of all PCRs was estimated to be between 98% and 102%. All assays were performed in triplicate for each sample. The comparative 2^−ΔΔ*C*t^ was applied for the relative fold changes of the target genes’ expression (*IGF-1R, IR, MYC*), normalized to an endogenous reference (*Hprt*) gene and a relevant control. ΔΔ*C*t is defined as the difference between the mean Δ*C*t of the treatment group and the control group, where Δ*C*t is the difference between the mean *C*t of the genes examined and the control genes in each sample. HPRT expression was considered as an endogenous control. The primers used are given in Table 1.

### 2.6. Wound Healing Assay

The effect of curcumin on migration was assessed by the wound healing assay. After the cells became 90% confluent, 5 µg/mL of mitomycin was added for 2 h to prevent proliferation. Then, the cells were scratched with a sterile 100 µL pipette tip, washed twice with PBS to remove debris, and finally, fresh media along with curcumin was added. Images of the wound gap were taken using an inverted microscope at 0, 24, and 48 h following scratching. All experiments were carried out in triplicate. “Measurement length” in ImageJ software was used to measure the distance between the wound edges.

### 2.7. Colony Formation Assay

The effect of curcumin on proliferation was determined by the colony formation assay. Cells were seeded in a 12-well plate at a density of about 40 cells/well. After 24 h of incubation, cells were treated with 20 µM of curcumin or DMSO as the vehicle. After 14 days of incubation, the plates were stained with 0.05% crystal violet (Sigma Chemical Company, St. Louis, MO, USA) for 2 h at room temperature, washed with tap water, air-dried, and the number of colonies were counted using a microscope. All experiments were performed in triplicate.

### 2.8. Statistical Analysis

Data are presented as the mean ± standard error (SE). Statistical analysis was performed using SPSS 19 software and determined by a two-tailed Student’s *t*-test. Differences were considered significant when *p* < 0.05. ImageJ software was used to measure the distance between the edges of the scratches. Figures were created using GraphPad Prism version 4 software (GraphPad Software, Inc., La Jolla, CA, USA). Additionally, the dose–response curve function of this software was used to determine the IC25 and IC50 doses.

## 3. Results

### 3.1. Isolation of 5-FU-Resistant SW480 Colon Cancer Cell Line (5FU-SW480)

**Figure 1:**
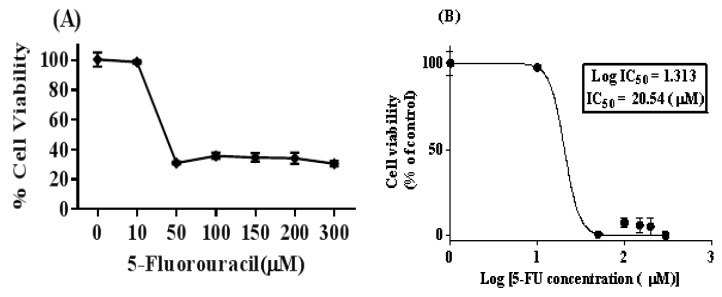
5-FU treatment in the SW480 colon cancer cell line leads to cell viability reduction. (**A**) The SW480 cell line was treated with different concentrations of 5-FU (0, 10, 50, 100, 150, 200, and 300 µM) for 72 h, and cell viability was evaluated using the MTT method. (**B**) The concentration–response curve was used to determine IC50 at a concentration of 20 µM, which indicated 50% growth inhibition. The results represent at least three independent experiments and are normalized to 100% of the control group, and presented as the mean ± standard error (SE) (95% confidence interval IC50 = 10.62–39.74).

The viability of the SW480 cells was calculated with the MTT assay after 72 h treatment with different concentrations of 5-FU (0, 10, 50, 100, 150, 200, and 300 µM). By using the dose–response curve ability of the GraphPad Prism 6 software, the IC50 of 5-FU was 20 µM. To provide 5FU-SW480 chemoresistant cells, we treated the 80% to 90% confluent monolayer of SW480 cells with a final concentration of 20 µM of 5-FU (IC50) for 72 h (Figure 1). Then, fresh working media was added and changed every other day and, after 10 days, the surviving chemoresistant cells, which we called 5FU-SW480, were used for the experiments.

### 3.2. Curcumin Exerted Cytotoxic Effects on Both SW480 and 5FU-SW480

**Figure 2:**
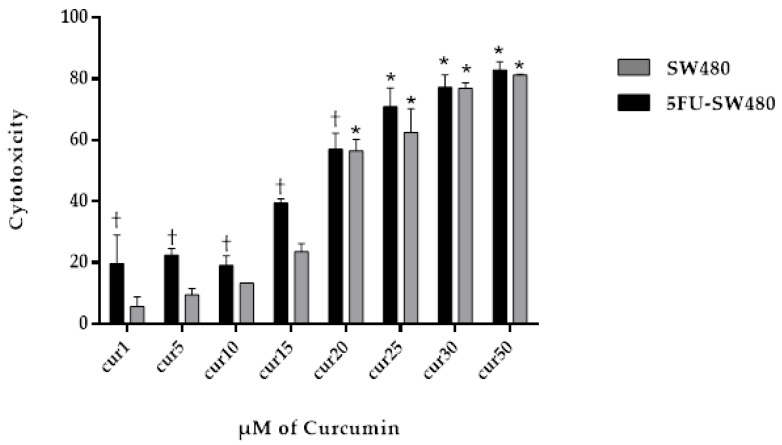
Effect of curcumin (CUR) on the viability of SW480 and 5-FU-treated cells. SW480 and 5FU-SW480 cells were treated with curcumin at different concentrations and their viability was determined by the MTT assay. Data are the average results of three independent experiments. †, * denote statistically significant difference (*p* < 0.05) when compared to the SW480 and control cells, respectively.

To test the cytotoxic effect of different concentrations of curcumin on SW480 and 5FU-SW480 resistant cells, they were exposed to several concentrations of curcumin for 72 h and then analyzed by the MTT assay. As shown in Figure 2, 72 h of treatment with curcumin promoted the inhibition of growth in both cells in a dose-dependent manner. By applying doses of 20–50 µM, this effect was significant in both cell lines (*p* < 0.001). Curcumin was more strongly effective on the 5FU-SW480 cells than the SW480 cells in lower doses (Figure 2). IC50 curcumin was calculated at 20.04 ± 0.96 µM for the SW480 cells and 17 ± 0.83 µM for 5FU-SW480 (Table 2).

### 3.3. Curcumin Induced Apoptosis in SW480 and 5FU-SW480

**Figure 3:**
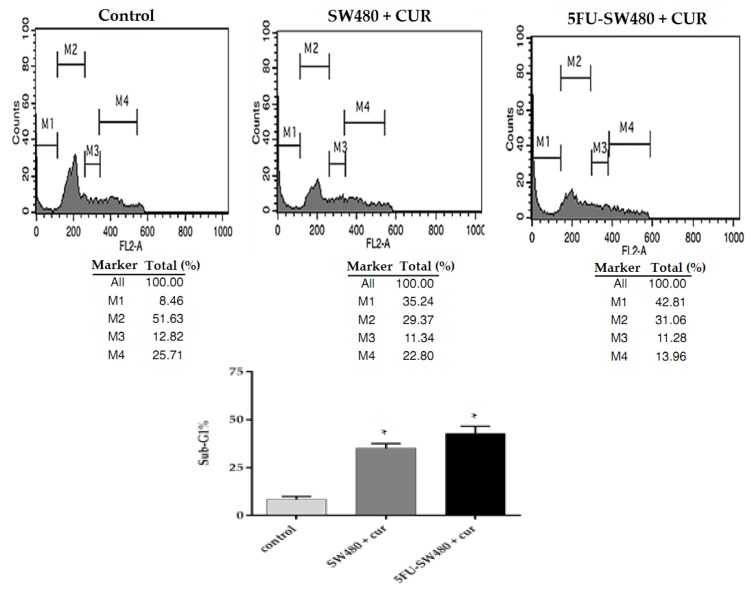
Evaluation of apoptosis by of SW480 and 5FU-SW480 PI flow cytometry. The cell lines (0.5–0.1 × 10^6^) were treated with curcumin (20 μM). Harvested cells stained with PI (50 mg/mL) were analyzed by FACS. The M1, M2, M3, and M4 bars show the sub-G0/G1, G0/G1, S, and G2/M phases, respectively. Each value represents the average of three independent experiments ± SE. * denotes a statistically significant difference (*p* < 0.05) compared to the control cells.

To determine the mechanism of growth inhibition induced by curcumin, we performed propidium iodide (PI) staining and cell cycle analysis by flow cytometry in SW480 and 5FU-SW480. Figure 3 shows that the incubation of SW480 and their 5-FU-resistant counterpart in curcumin (20 µM for 72 h) increased the percentage of cells in the sub-G1 phase (*p* ˂ 0.05). It seems that the percentage of 5FU-SW480 was slightly more than that in the SW480 cells, however, the differences were not statistically significant.

### 3.4. Colony Formation of SW480 and 5FU-SW480 was Decreased by Curcumin

**Figure 4:**
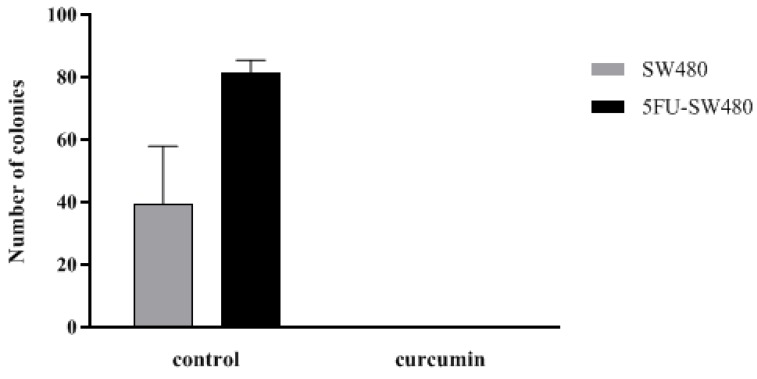
Effect of curcumin efficiency in preventing colony formation of SW480 and 5FU-SW480 cells. Cell suspensions (40 cells/well) were seeded onto 12-well plates. The number of colonies in each well of the plate was counted with an inverted microscope after two weeks of culture. Each value is presented as the mean ± SE of three independent experiments.

To investigate the proliferation capacity of the cells, the effect of curcumin on the clonogenic survival of SW480 cells and their 5-FU-resistant counterparts were examined. Interestingly, curcumin (20 µM, 14 days) markedly prevented the ability of both the SW480 cells and 5FU-SW480 to form colonies (Figure 4). These results indicate that curcumin, at a concentration of 20 µM, had antiproliferative and antitumor effects on both SW480 and chemoresistant 5FU-SW480 cells.

### 3.5. Curcumin Decreased Cell Migration in SW480 and 5FU-SW480

**Figure 5:**
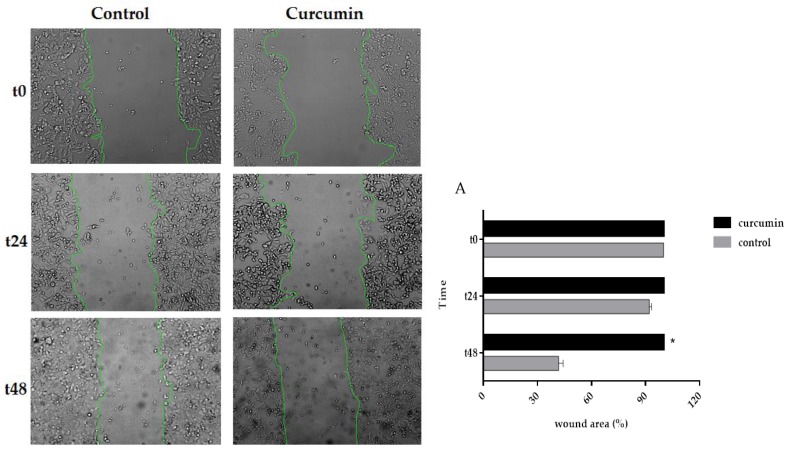

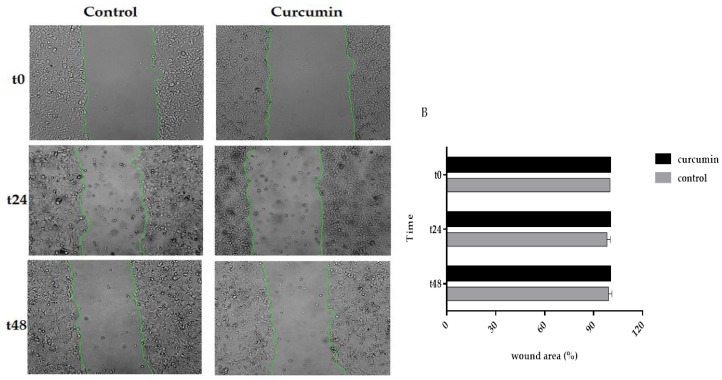
Effects of curcumin on the motility of the 5FU-SW480 and SW480 cells. The migration of the 5FU-SW480 (**A**) and SW480 (**B**) cells was assessed by the wound healing scratch assay of 20 μM curcumin when compared to the control group. Representative images of wound closure were taken at 0, 24, and 48 h after injury under 40 × magnification. Each value is presented as the mean ± SE of three independent experiments.

As curcumin inhibited the cell growth of colon cancer cell lines, we asked whether cell migration could be affected by this natural polyphenol. To do this, the cells were treated with DMSO, as the vehicle, or curcumin (20 µM) for 24 and 48 h in a scratch assay. Then, the wound areas were evaluated, and images were taken under an inverted microscope. As shown in Figure 5, there was a marked decrease in the edge closure speed of the wound in curcumin-treated cells, specifically, in the 5FU-SW480 cells. The difference in migration following 48 h of curcumin exposure was statistically significant in 5FU-SW480 in comparison to the untreated cells.

### 3.6. Curcumin Decreased Expression of Insulin and IGF-1 Receptors in Addition to MYC

**Figure 6:**
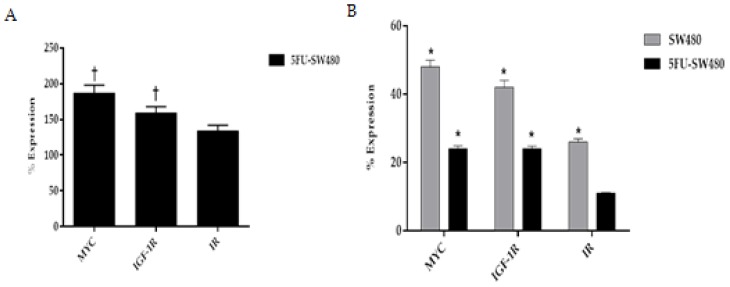
Real-time PCR of *MYC*, *IGF-1R*, and *IR* gene expression in the SW480 and 5FU-SW480 cells. (**A**) Basal expression of genes in the 5FU-SW480 cells compared to the SW480 cells. (**B**) Comparing the effect of 48 h treatment with 20 μM curcumin on the expression of all three genes in the 5FU-SW480 and SW480 cells. All values are presented as the mean ± SE of three independent experiments in triplicate. †, * denotes a statistically significant difference (*p* < 0.05) when compared to the SW480 and control cells, respectively.

To investigate the anticancer mechanism of curcumin, the expression of *insulin* and *IGF-1 receptors* as well as a *MYC* oncogene were analyzed using qRT-PCR. We first assessed the pattern of expression of *MYC*, *insulin*, and *IGF-1 receptors* in 5-FU chemoresistant cells and then compared it with SW480. This comparison revealed that the expression of *MYC*, *insulin*, and *IGF-1R* in 5-FU chemoresistant cells was significantly higher than that of the SW480 cells (Figure 6A). The results of the present study showed that incubation of SW480 and 5FU-SW480 by curcumin (20 µM for 48 h) reduced the expression of the *insulin* and *IGF-1 receptors* mRNA in comparison to the DMSO-treated cells (Figure 6B). Curcumin inhibitory activity on the expression the *insulin* and *IGF-1 receptors* in 5FU-SW480 were markedly more obvious than in SW480 cells. Although *MYC* expression also decreased in both cells treated by 20 µM curcumin for 48 h, curcumin-induced attenuation of *MYC* was more effective in the 5FU-SW480 cells in comparison to SW480.

## 4. Discussion

The current study was carried out to suggest the anti-metastatic effect of curcumin as a new adjuvant therapeutic for colorectal cancer. We found that curcumin reduced the expression of *MYC*, *insulin*, and *IGF-1 receptors*. Additionally, curcumin markedly decreased the proliferation and migration of 5-FU-resistant SW480 cells when compared to the SW480 control cells.

Previously, preclinical studies demonstrated that curcumin prevented the migration of colon cancer cells. Curcumin inhibits the migration of human colon cancer COLO205 cells through the inhibition of NFκB [22]. Most studies have emphasized the anti-inflammatory action of curcumin in the anti-metastatic effect of curcumin [23].

According to CSC theory, a subpopulation of tumor cells are responsible for tumor heterogeneity and resistance to conventional therapy [9]. The ability of these cells to migrate and become dormant in unsuitable microenvironments makes them a major suspect in cancer metastasis and recurrence. It has been shown that inflammation pathways, notably, the formation of proinflammatory cytokines induced by NFκB, are one the driving forces of metastasis in CSCs [24].

A comparison of 5-FU and SW480 cells indicated that *MYC*, *insulin*, and *IGF-1 receptors* were significantly more expressed in 5-FU chemoresistant cells. This is in accordance with Codony-Servat et al., who reported that chemotherapy-resistant cells and pretreated metastasis colorectal cancer paired biopsies revealed an increase in the nuclear expression of *IGF-1R* in comparison with sensitive cell lines and untreated patients [25]. Consequently, in our pursuit to identify agents that may reduce the resistance of cancer cells to treatment, we examined the effects of curcumin on inducing the downregulation of the mentioned genes. New evidence supports a role for IR and IGF-1R dysregulation in intestinal stem cells, progenitors, differentiated epithelium, and tumors [26]. One of the major pathways involved in colorectal cancer development and progression is IGF-1R [27]. Dallas and colleagues used an oxaliplatin-resistant colon cancer model and showed that the suppression of cancer cell development and progression could be achieved by IGF-1R inhibition [28]. Treatment with curcumin, as an adjuvant intervention to a chemotherapeutic regimen named FOLOX, resulted in cancer cell apoptosis through downregulating the *epidermal growth factor receptor (EGFR*) and *IGF-1R* expression. It seems that the inhibitory effect of curcumin on IGF-1R is induced by stimulating the expression of its inhibitor, IGF-binding protein-3, IGFBP3 [27,29]. In addition, Codony-Servat et al. revealed that curcumin could interfere with the nuclear localization of IGF-1R. The proposed mechanism is the interaction of curcumin with PIASS, which is a STAT3-inhibitor protein and thereby leads to the inhibition of STAT activity [25]. Thus, targeting the IGF-1R/IGF-1 signaling pathway can be considered as an effective approach to both colorectal cancer prevention and treatment. In support of the role of IR and IGF-1R in metastasis, Lu et al. showed that insulin promoted the proliferation and migration capacity of HCT-116 via IRS-1 and PI3K/AKT and mitogen-activated protein kinase (MAPK) signaling [19]. Some studies have demonstrated that *IR* and *IGF-1R* are overexpressed in cancer stem cells. Our results also suggest that SW480 cells resistant to 5-FU expressed more *IR* and *IGF-1R* mRNA.

Researchers in a study revealed that IR was downregulated by curcumin and vitamin D3 administration in streptozotocin-induced diabetic rats [20]. The results of our study showed that curcumin suppresses IR and IGF-1R in both SW480 and 5FU-SW480. To the best of our knowledge, these results provide evidence, for the first time, that curcumin may influence the proliferation and migration capacity of cancer cells through targeting IR and IGF-1R signaling.

In vivo studies have stated that the expression of *MYC* in the cancerous tissues of patients who experienced recurrence, following 5FU-based adjuvant chemotherapy, was significantly higher than those without recurrence [30]. *MYC* overexpression has also been shown in resistant cancer cells, which confirms these findings [31,32]. Some evidence has demonstrated that the MYC oncogene also plays a crucial role in the stemness traits, especially self-renewal and multilineage differentiation, of CSCs and their migration ability. MYC regulates the epithelial-to-mesenchymal transition (EMT), which has a pivotal role in the promotion of stemness and the migration of CSCs [33]. The results of the present experiment contrasted with a previous study demonstrating that difluorinated-curcumin, a curcumin analogue, could significantly inhibit *MYC* expression in chemoresistant colon cancer cells, while curcumin could not exert such an effect [34]. It has been previously shown that curcumin promotes apoptosis through the downregulation of MYC and other antiapoptotic pathways including bcl-2 and the potentiation of proapoptotic signals such as p53 and p21 [35,36].

## 5. Conclusions

In summary, the results from the current study show that curcumin inhibits the cell proliferation and migration of chemoresistant colorectal cancer cells, along with a marked attenuation of *IR*, *IGF-1R*, and *MYC* expression. These results suggest the possible therapeutic effectiveness of curcumin in adjuvant therapy in metastatic colorectal cancer.

## Figures and Tables

**Table 1:** Details of the primer pairs used in this study.

Gene	Primer Sequence 5′-3′
MYC	F: AGCGACTCTGAGGAGGAAC R: GCTGCGTAGTTGTGCTGATG
IGF-1R	F: AGAAGGAGGAGGCTGAATAC R: GGTCGGTGATGTTGTAGGT
IR	F: TAGAAGGCGAGAAGACCATC R: GTGACACCAGAGCGTAGG
HPRT	F: CCTGGCGTCGTGATTAGTGA R: AAGACGTTCAGTCCTGTCCAT

**Table 2:** Inhibition concentrations of curcumin (CUR) on the SW480 and 5FU-SW480 cells. The values demonstrate the inhibition concentrations (IC25, IC50) (µM) of curcumin after 72 h treatment. Data are the average results of three independent experiments.

Treatment (CUR)	IC25	IC50
SW480	10.75 ± 0.53	20.04 ± 0.96
5FU-SW480	6.15 ± 1.34	17.56 ± 0.83
